# Silencing of HSulf-2 expression in MCF10DCIS.com cells attenuate ductal carcinoma in situ progression to invasive ductal carcinoma *in vivo*

**DOI:** 10.1186/bcr3140

**Published:** 2012-03-12

**Authors:** Ashwani Khurana, Hiedi McKean, Hyunseok Kim, Sung-Hoon Kim, Jacie Mcguire, Lewis R Roberts, Matthew P Goetz, Viji Shridhar

**Affiliations:** 1 Department of Experimental Pathology, Mayo Clinic College of Medicine, 200 First Street SW, Rochester, MN 55905, USA; 2 Cancer Preventive Material Development Research Center (CPMDRC) and Institute, College of Oriental Medicine, Kyunghee University, Dongdaemun-gu, Seoul 130-701, South Korea; 3 Section of Gastroenterology, Mayo Clinic College of Medicine, 200 First Street SW, Rochester, MN 55905, USA; 4 Department of Oncology, Mayo Clinic College of Medicine, 200 First Street SW, Rochester, MN 55905, USA

## Abstract

**Introduction:**

Ductal carcinoma *in situ *(DCIS) of the breast is a heterogeneous group of proliferative cellular lesions that have the potential to become invasive. Very little is known about the molecular alterations involved in the progression from DCIS to invasive ductal carcinoma (IDC). Heparan endosulfatase (HSulf-2) edits sulfate moieties on heparan sulfate proteoglycans (HSPGs) and has been implicated in modulating heparin binding growth factor signaling, angiogenesis and tumorigenesis. However, the role of HSulf-2 in breast cancer progression is poorly understood. MCF10DCIS.com cells (referred as MCF10DCIS) express HSulf-2 and form comedo type DCIS and progress to IDC when transplanted in immune-deficient mice and, therefore, is an ideal model to study breast cancer progression. We evaluated the role of HSulf-2 in progression from DCIS to IDC using mouse fat pad mammary xenografts.

**Methods:**

Non-target control (NTC) and HSulf-2 knockdown in MCF10DCIS breast cancer cells were achieved by NTC shRNA and two different lentiviral shRNA against HSulf-2 respectively. Xenografts were established by injecting NTC and HSulf-2 deficient MCF10DCIS cells in mouse mammary fat pads. Xenografts were subjected to H&E staining for morphological analysis, TUNEL and Propidium iodide staining (to determine the extent of apoptosis), Western blot analysis and zymography.

**Results:**

Using a mouse mammary fat pad derived xenograft model, we observed that compared to control treated xenografts, down-regulation of HSulf-2 was associated with significant delays in growth at Week 7 (*P-*value < 0.05). Histological examination of the tumors demonstrated substantial differences in comedo necrosis, with marked luminal apoptosis and up-regulation of apoptotic markers Bim, cleaved PARP and cleaved caspase 3 in HSulf-2 depleted xenografts. Furthermore, HSulf-2 depleted xenografts retained the basement membrane integrity with decreased activity and expression of matrix metalloproteinase 9 (MMP-9), an enzyme critical for degradation of extracellular matrix compared to nontargeted control.

**Conclusion:**

Our data suggest that HSulf-2 expression may be critical for human breast cancer progression. Down-regulation of HSulf-2 leads to retention of comedo type DCIS and delays the progression of DCIS to IDC. Further studies are necessary to determine if therapeutic targeting of HSulf-2 expression might delay the progression of DCIS to IDC.

## Introduction

Ductal carcinoma *in situ *(DCIS) consists of proliferating malignant clonal cells within the lumen of mammary ducts with no evidence of invasion through the basement membrane into surrounding stroma [1]. While it is generally accepted that nearly all invasive breast carcinomas arise from DCIS [2], few patients with DCIS will develop invasive breast cancer after standard treatments, such as surgery, radiation and tamoxifen [3,4]. While increasing data suggest that epithelial mesenchymal transition (EMT), a process characterized by activation of matrix metalloproteinase (MMP) enzymes involved in the degradation of extracellular matrix [5,6] and the acquisition of invasive phenotype, is often associated with progression of DCIS to IDC [7,8], the molecular events underlying EMT are poorly understood. Molecular markers, which are associated with the process of transition from DCIS to invasive ductal carcinoma (IDC), may allow clinicians and patients to forgo more aggressive therapies, such as mastectomy. In order to understand the progression of DCIS to IDC, several reports have determined the alteration in genetic (intrinsic) and stromal (extrinsic) associated with DCIS and IDC [9-11].

Heparan sulfate proteoglycans (HSPGs) serve as co-receptors for many heparin binding growth factor receptors [12,13]. HS is highly sulfated and is desulfated at 6-O sulfate moiety by two HS editing enzymes known as heparan sulfatases 1 and -2 [14]. Catalytically, these enzymes desulfate the sulfation moieties on the HSPGs and this action disrupts the ternary complex formation between heparin binding ligands, such as bFGF2 and its cognate receptor, FGFR2 and co-receptor HSPGs [13]. Similarly, various heparin-binding growth factors signaling have been shown to be up-regulated in breast cancer tumorigenesis and are remodeled by a group of enzymes known as heparan sulfatases [15-21].

HSulfs have been shown to promote wingless type (Wnt) signaling known to promote cancer growth [22]. Previous reports indicate that HSulf-2 has both tumor suppressing and tumor promoting roles in cancer [23,24]. More specifically, studies have indicated that HSulf-2 is the most frequently, differentially-expressed gene between ductal carcinoma *in situ *and invasive ductal carcinoma [25]. The tumor promoting functions of HSulf-2 have been supported by previous reports suggesting HSulf-2 as a positive regulator of Wnt pathway in pancreatic cancer cells [22]. Furthermore, it has been shown that HSulf-2 has a pro-angiogenic role in breast cancer [23]; however, more recent data suggest that HSulf-2 attenuates metastasis [24]. Although HSulf-2's role in cancer has been investigated in various tumor types, the precise role of HSulf-2 in breast cancer tumorigenesis is not clearly defined. In the present study, we have determined the role of HSulf-2 in progression of ductal carcinoma *in situ *to invasive ductal carcinoma using the MCF10DCIS cell line. Increased proliferation of epithelial cells, loss of acinar organization and filling of the luminal space has been shown in the MCF10DCIS model, a well-characterized xenograft progression model of DCIS to IDC [26]. Our study suggests that HSulf-2 might play an important role in the transition from DCIS to an invasive phenotype (IDC). HSulf-2 promotes basement membrane proteolysis via up-regulation of MMP-9 activity and promotes progression of DCIS to IDC thus opening avenues to therapeutically target HSulf-2.

## Materials and methods

### Cell lines and cell culture

Breast cancer cell lines MCF10DCIS and MCF10AT1 were grown as described previously [16,27,28]. Anti-HSulf-2 antibody was a gift from Dr Lewis Roberts (Mayo Clinic, Rochester, MN, USA). Antibodies used in these studies are anti-α-tubulin (Sigma, St. Louis, MO, USA), anti-Bnip3, anti-Bim EL, anti-cleaved PARP, anti- cleaved caspase 3, (Cell Signaling, Boston, MA, USA) anti-MMP-2, anti-MMP-9 and anti-MMP-14 antibodies (Chemicon, Billerica, MA, USA).

### Western immunoblot

Equal amounts of proteins from the cells were resolved on SDS-PAGE followed by transfer on PVDF membrane and immuno-probed with indicated antibodies as previously described [29].

### Small interfering RNA transfections and shRNAs

Short-hairpin RNAs (shRNAs) cloned into the lentivirus vector pLKO.1-puro were chosen from the human library (MISSION TRC-Hs 1.0) and purchased as glycerol stock from Sigma. The control shRNA (non-target shRNA vector, Sigma) contains a hairpin insert that will generate siRNAs but contains five base pair mismatches to any known human gene. Target sequence for HSulf-2 shRNA (HW11) CAAGGGTTACAAGCAGTGTAA and HSulf-2 shRNA (HW13): CCACAACACCTACACCAACAA. Lentivirus particles were produced by transient transfection of two different plasmids targeting HSulf-2 (pLKO.1-HSulf-2) and pLKO.1 non-target control (NTC) along with packaging vectors (pVSV-G and pGag/pol) in 293T cells as previously described [30,31].

### Mouse mammary fat pad injections

MCF10DCIS xenografts were generated by injecting MCF10DCIS cells stably expressing non-targeted control and HSulf-2 knockdown clones HW11 and HW13. A total of 1.0 × 10^6 ^cells in 0.1 ml of matrigel were subcutaneously injected at each nipple of gland #5 of female nude mice. Xenografts were removed at Weeks 3, 5 and 7, either fixed in formalin buffer or frozen immediately in liquid nitrogen or stored at -80°C. All animal work was conducted under protocols approved by the Mayo Clinic Institutional Animal Care and Use Committee and the animals were housed in institutional animal facilities. Tumor volume was calculated with the formula *V *= 1/2 *a *× *b*^2^, where "*a" *is the longest tumor axis, and "*b" *is the shortest tumor axis.

### Immunohistochemistry

Each specimen of xenografts obtained from NTC and HSulf-2 down-regulated clones were stained with H&E for morphological analysis. For immunohistochemistry, xenografts embedded in paraffin were cut at 5 to 7 μm, mounted on glass and dried overnight at 37°C. All sections were deparaffinized in xylene, rehydrated through a graded alcohol series and washed in phosphate-buffered saline (PBS). PBS was used for all subsequent washes and for antiserum dilution. Tissue sections were quenched sequentially in 3% hydrogen peroxide in aqueous solution and blocked with 6% non-fat dry milk in PBS for 1 h at room temperature. Slides then were incubated at 4°C overnight with a rabbit polyclonal antiserum specific for HSulf-2 at a final 1:100 dilution and SMA at a final 1:100 dilution (Dako, Cat # M0851, Glostrup, Denmark) in PBS-3% non-fat dry milk. After three washes in PBS to remove the excess antiserum, the slides were incubated with diluted goat anti-rabbit biotinylated antibody (Vector Laboratories, Burlingame, CA, USA) at 1:200 dilution in PBS-3% non-fat dry milk for 1 h. All the slides were then processed by the ABC method (Vector Laboratories, Burlingame, CA, USA) for 30 minutes at room temperature. Diamonibenzidine (Vector Laboratories) was used as the final chromogen and hematoxylin was used as the nuclear counterstain. Negative controls for each tissue section were prepared by leaving out the primary antiserum.

### Immunofluoresence

For immunofluoresence, similar steps were followed as described for immunohistochemistry till incubation with primary respective antibodies. After three washes in Tris-**Buffered **Saline and Tween 20 to remove the excess antiserum, the slides were incubated with diluted anti-rabbit - Fluorescein Isothiocyanate and anti-mouse TRITC antibody. Slides were finally mounted on mounting medium with 4',6-diamidino-2-phenylindole (DAPI) for nuclear staining or propidium iodide was added prior to mounting on mounting medium. No primary antibody was added in negative controls.

### Terminal deoxynucleotidyl transferase biotin-dUTP nick end-labeling (TUNEL) assay

TdT-mediated dUTP nick end labeling assay was used to detect apoptosis in xenografts obtained from NTC and HSulf-2 downregulated HW11 and HW13 clones as recommended by the manufacturer of APO Tag kit (Millipore Corporate, Billerica, MA, USA).

### Gelatin zymography

MMP-2 and MMP-9 enzymatic activity in mouse derived xenografts was performed by SDS-PAGE gelatin zymography. Gelatinases present in the tissue lysates degrade gelatin in the SDS-PAGE leaving a clear white band after commassie staining of the gel. Tissue samples were homogenized in the lysis buffer. Equal protein was denatured in the absence of reducing agent and electrophoresis in 7.5% SDS-PAGE containing 0.1% (w/v) gelatin. The gel was incubated in the presence of 2.5% Triton X-100 at room temperature for two hours and subsequently at 37°C over might in 10 mM CaCl2, 0.15 M NaCl, and 50 mM Tris (pH 7.5). The gel was stained with 0.25% Coomassie Blue.

## Results

### HSulf-2 downregulation attenuates tumor growth *in vivo*

**Figure 1:**
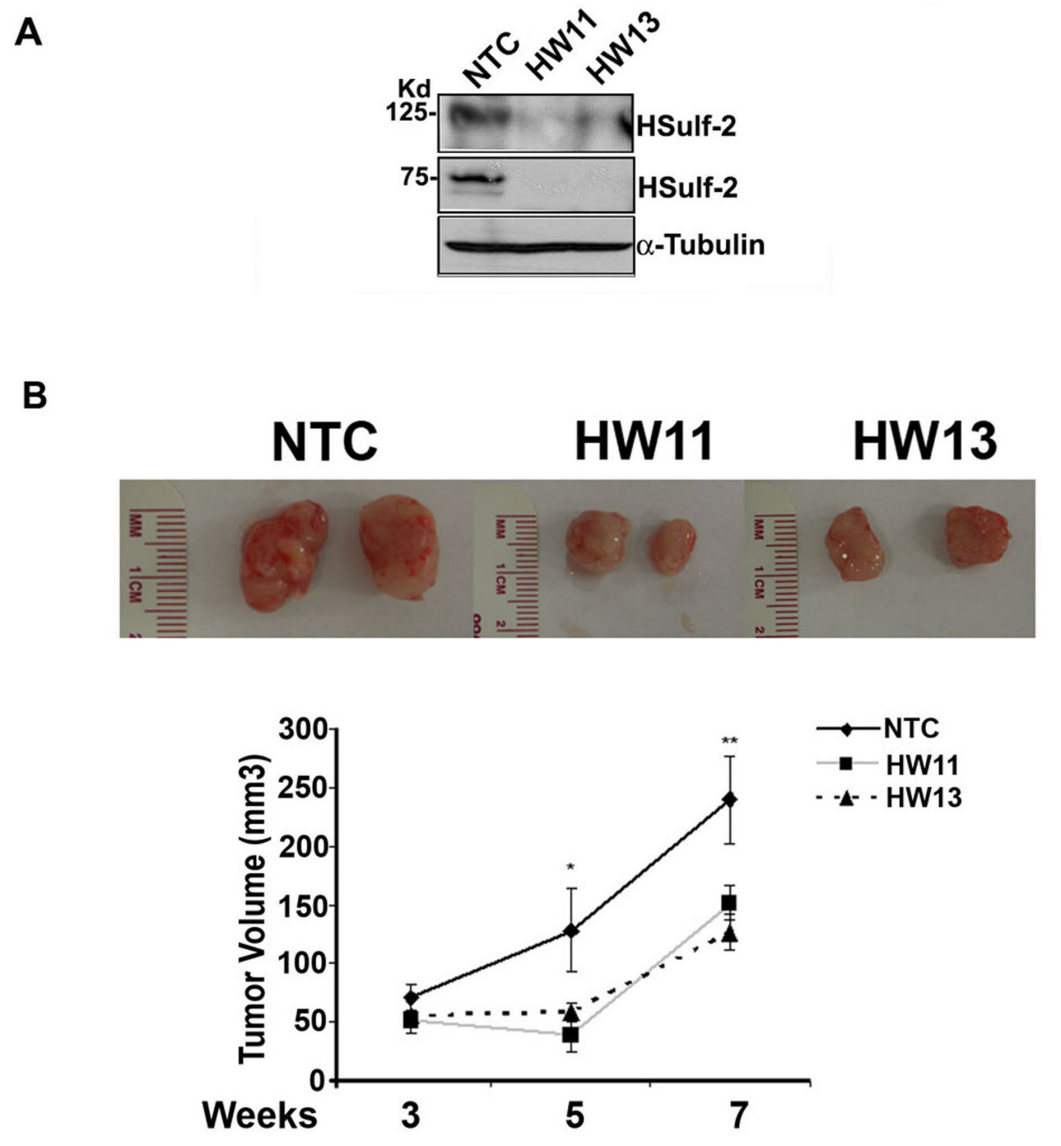
**HSulf-2 knockdown attenuates tumor growth**. **(A) **Western immunoblot analysis using anti-HSulf-2 and anti-tubulin antibodies shows efficient down-regulation of HSulf-2 in HW11 and 13 compared to NTC cells. **(B) **NTC and HSulf-2 depleted clones were injected in mouse mammary fat pad as described in Materials and methods. Tumor growth was monitored at Weeks 3, 5 and 7. Eight mice per group were evaluated. Inset: Excised tumors from NTC and HSulf-2 depleted clones. Graphs show quantitation of the tumor volume at indicated intervals among the groups. *P-*value < 0.05 (Student *t *test analysis).

To evaluate the role of HSulf-2 in breast cancer, we generated batch stable clones with two different viral shRNAs, HW11 and HW13 targeted to different regions on HSulf-2 mRNA in MCF10DCIS cells as described in Materials and methods. Western immunoblot analysis (Figure 1A) shows robust HSulf-2 down-regulation in these batch clones. Non-targeted control (NTC) ShRNA served as control.

To gain insights into the role of HSulf-2 in the progression from DCIS to IDC *in vivo*, NTC and HSulf-2 down-regulated batch stable clones HW11 and HW13 in MCF10DCIS cells were injected into mammary fat pad as described in Materials and methods. Tumor tissues were excised at the indicated intervals and either immediately frozen or saved in fixative. Tumor growth was monitored by caliper measurements at Weeks 3, 5 and 7 (Figure 1B). As shown in Figure 1B, tumor growth in both HSulf-2 down-regulated clones (HW11 and HW13) were attenuated compared to NTC cells (*P-value < 0.05, **P-value < 0.05). These data suggest that depletion of HSulf-2 results in decreased tumor growth.

### HSulf-2 knockdown delays ductal carcinoma in-situ (DCIS) to invasive ductal carcinoma

**Figure 2:**
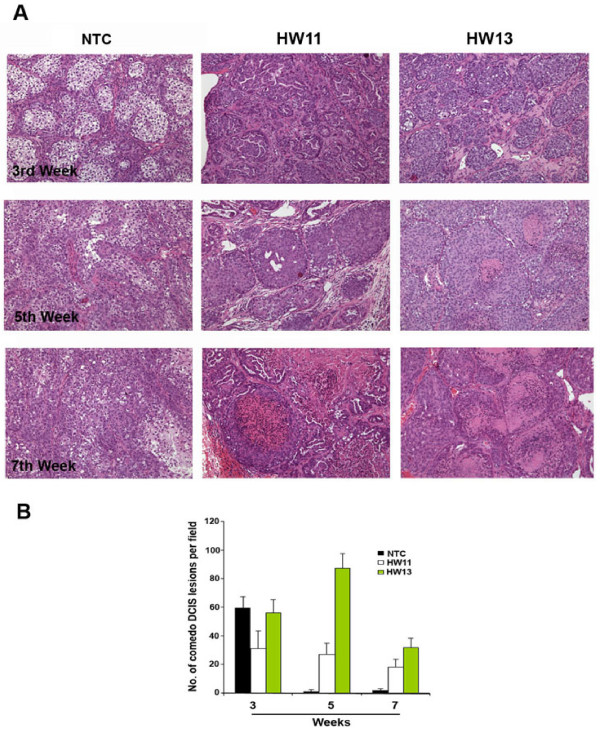
**(A) HSulf-2 knockdown xenografts retains comedo structures for longer duration**. H&E staining of NTC and HSulf-2 depleted clones derived xenografts at Weeks 3, 5 and 7. **(B) **Quantitation of intact comedo structures in NTC and HSulf-2 depleted clones as shown in Figure 2A. At least six different slides per sample were evaluated. *P-*value < 0.05.

Histopathological evaluation of xenografts by H&E staining clearly showed comedo type lesions with well formed basement membrane surrounding the ductal lesions at Week 3 in NTC clone and in xenografts derived from HW11 and HW13 clones (Figure 2, top panel). While HSulf-2 depleted HW11 and HW13 xenografts exhibited an increasing number of ductal lesions with defined basement membrane at Weeks 5 and 7, the NTC derived xenografts exhibited a more invasive phenotype with less defined DCIS structures (Figure 2A, middle and bottom panels). Graphical representation of the number of comedo DCIS structures in these clonal lines are shown in Figure 2B for the period of the experiment and demonstrate significantly more comedo type lesions in the HSulf-2 depleted clones compared to the control treated.

### HSulf-2 is expressed in comedo structures formed *in vivo*

**Figure 3:**
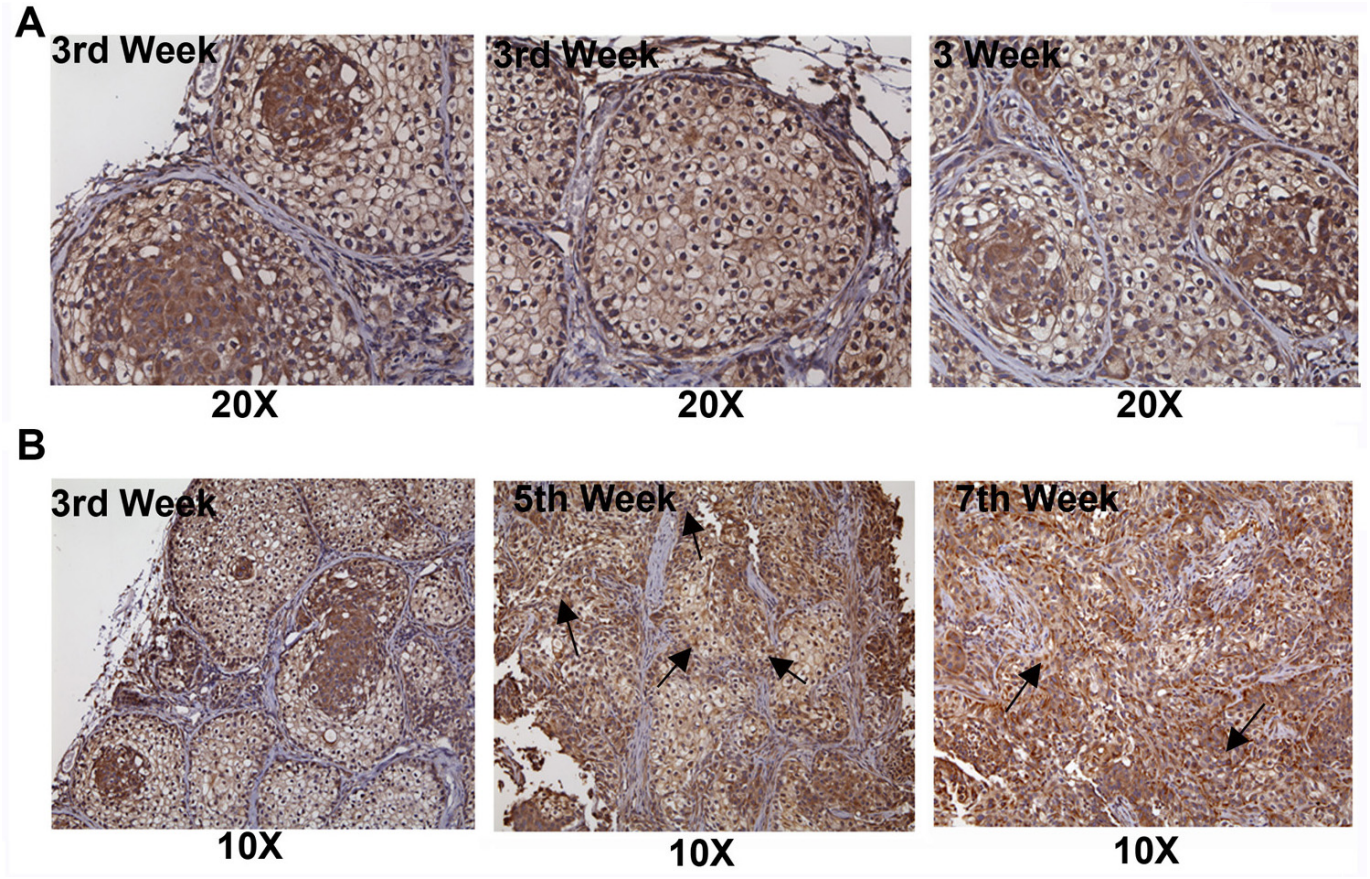
**HSulf-2 is expressed in ductal lesions formed *in vivo *in NTC derived xenografts**. **(A) **Immunohistochemistry analysis revealed expression of HSulf-2 in central areas as well as areas surrounding basement membrane. Limited staining of HSulf-2 was observed in stromal fraction. (Magnification 20×). **(B) **Immunohistochemistry analysis of HSulf-2 expression in NTC at Weeks 3, 5 and 7 (Magnification 10×).

**Figure 4:**
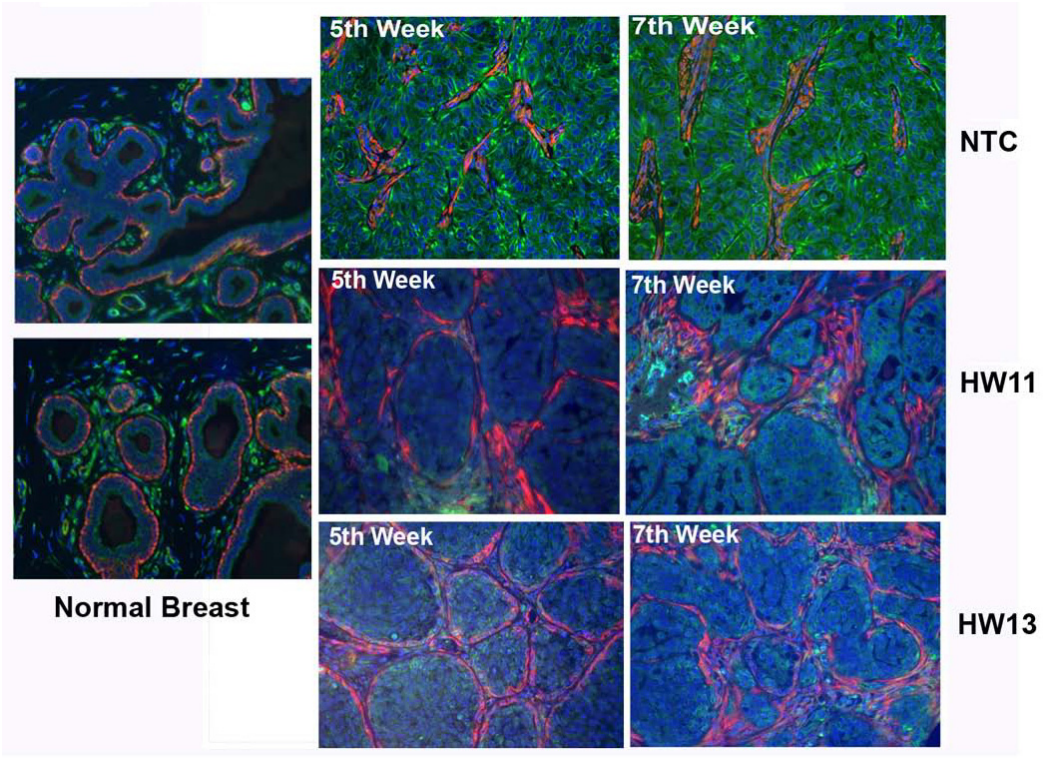
**HSulf-2 depleted xenografts retain ductal structures**. HSulf-2 (green) and smooth muscle actin (red) is detected by immunofluresence microscopy in normal human breast and mouse-derived xenografts at Weeks 5 and 7 in NTC and HSulf-2 depleted xenografts. (Magnification 20×).

To understand the pattern and extent of HSulf-2 expression in MCF10DCIS derived NTC xenografts, we evaluated HSulf-2 staining in xenografts obtained from NTC clones by immunohistochemistry as described in Materials and methods. HSulf-2 was expressed predominantly in ductal lesions and in the central filled lumen areas with least expression in stroma at Week 3 (Figure 3A). Increased expression of HSulf-2 staining was evident as the DCIS structures evolved into IDC with basement membrane dissolution (Figure 3B - Arrows). Similarly, immunofluoresence microscopy with SMA staining (marker of myoepithelial layer) of the xenografts (Figure 4) indicated integrity of basement membrane around the ductal lesions (TRITC labeled SMA). Notably, HSulf-2 expression was predominant in the stromal compartment in normal breast tissue whereas HSulf-2 was highly expressed in epithelial cells of comedo structures apparent in NTC derived xenografts. Furthermore, HSulf-2 depleted xenografts show well preserved basement membrane, whereas NTC derived xenografts show patches of basement membrane indicating its disruption at Weeks 5 and 7. Nearly absent staining was observed with anti-HSulf-2 antibody in these xenografts indicating HSulf-2 depletion was also maintained during the breast cancer progression at indicated time intervals (Figure 4).

### Enhanced luminal apoptosis of ductal lesions marks HSulf-2 depleted xenografts

**Figure 5:**
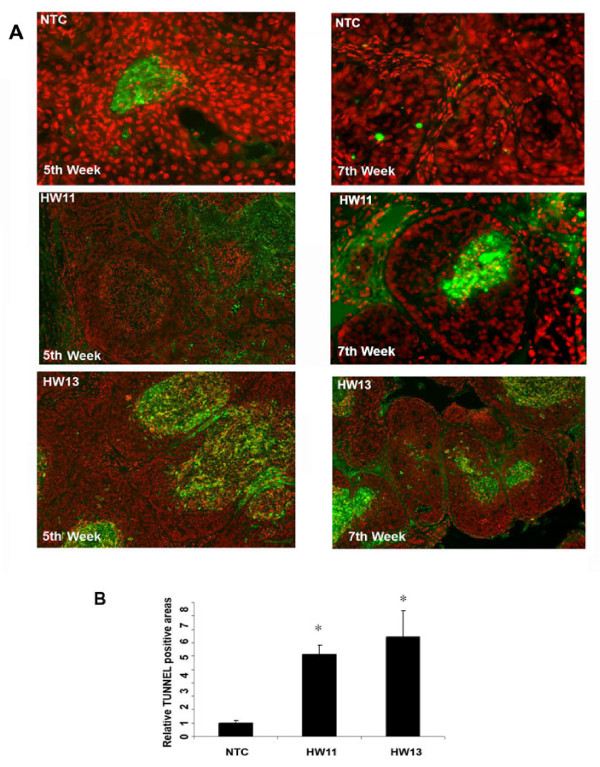
**HSulf-2 knockdown promotes marked apoptosis *in vivo***. **(A) **TUNEL staining(green) and nuclei staining with propidium iodide (red) were performed as described in Materials and methods in NTC and HSulf-2 depleted clones at Weeks 5 and 7. **(B) **Quantitation of apoptotic areas (comedo lesions) in NTC and HSulf-2 depleted clones (HW11 and HW13). At least 100 fields were examined in 6 different sections of each group. *P-value < 0.05, Student *t *test analysis.

Our *in vivo *data clearly suggest that HSulf-2 depletion resulted in decreased tumor volume and increased necrotic areas in H&E staining (Figure 2). Therefore, we further evaluated the effect of HSulf-2 knockdown on apoptosis in NTC and HSulf-2 depleted xenografts. To analyze the extent of apoptosis in xenografts, we performed TUNEL and propidium iodide (PI) staining on xenografts as described in the Materials and methods section. Our data revealed that HSulf-2 depletion resulted in a higher degree of apoptotic positive areas (TUNEL positive, FITC labeled) in the xenografts as compared to NTC at Week 5 and notably at Week 7 (Figure 5A, panel 1). More importantly, xenografts derived from HSulf-2-depleted clones showed marked TUNEL positive ductal lesions with intact basement membrane even at Weeks 5 and 7 (Figure 5, panels 2 and 3). Figure 5B shows quantification of these data by bar graph (*P-value < 0.05, t test). At least 100 fields were examined in six different sections of each group.

**Figure 6:**
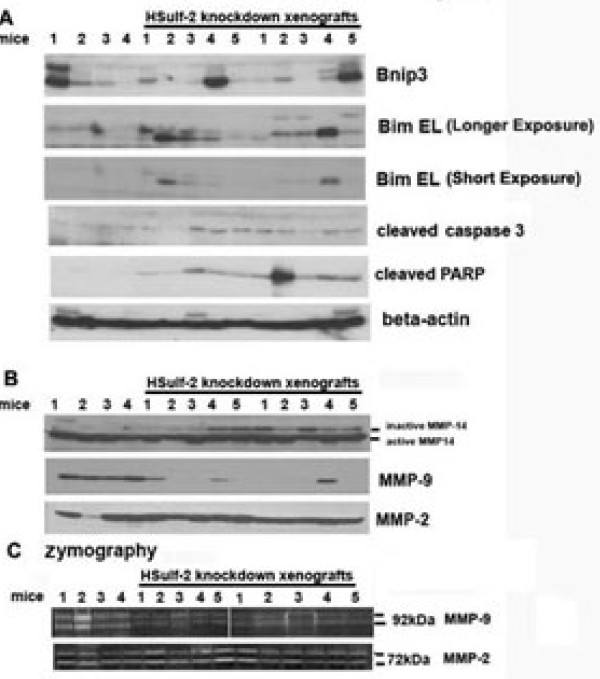
**Analysis of NTC and HSulf-2 xenografts**. **(A) **NTC (four mice) and HSulf-2 derived (five mice/clones) xenograft lysates were subjected to Western blot analysis using antibodies against Bnip3, Bim EL, cleaved caspase 3, cleaved PARP, beta-actin, and **(B) **with anti-MMP-14, MMP-9 and MMP-2 antibodies. **(C) **NTC and HSulf-2 depleted xenografts were subjected to gelatin zymography as described in the Materials and methods to detect activities of MMP-2 and MMP-9.

To further identify key players involved in apoptotic cell death and basement membrane remodeling, we extended our investigation in lysates derived from xenografts as described in Materials and methods. Western blot analysis of lysates derived from xenografts suggest that pro-apoptotic proteins Bim, cleaved PARP, cleaved caspase 3 were up-regulated in HSulf-2 depleted xenografts (four to five mice/group) with very little change in Bnip3 expression compared to NTC derived xenografts (Figure 6A). These data suggest that HSulf-2 knockdown resulted in increased apoptosis in the center of ductal lesions.

### HSulf-2 knockdown attenuates MMP-9 expression in mouse xenografts and MCF10DCIS cells

We noted two major effects of HSulf-2 depletion on mouse derived xenografts: a) increased luminal apoptosis and b) decreased basement membrane breakdown. Our observation that HSulf-2 knockdown resulted in decreased breakdown of basement membrane even at Week 7 of tumor growth indicated that HSulf-2 presence might be critical for basement membrane (BM) breakdown. Breakdown of BM reflects transition from ductal to invasive ductal carcinoma [32]. This disintegration of basement membrane layer surrounding the ductal lesions can be attributed to high activity of MMPs. Therefore, we next evaluated the effect of HSulf-2 on MMP expression levels. Our analysis on NTC and HSulf-2 depleted clones reveals that HSulf-2 depletion did not alter MMP-2 and -14 expression (although slight accumulation of inactive form of MMP-14 was observed in HSulf-2 depleted xenografts), whereas marked reduction of MMP-9 expression was observed in HSulf-2 depleted xenografts as compared to NTC (Figure 6B). As shown in Figure 6C, gelatin zymography of NTC derived xenografts showed enhanced gelatin degradation as compared to HSulf-2 depleted xenografts, whereas no change in MMP-2 activation was observed. These data suggest that HSulf-2 depletion has negative impact on MMP-9 expression and, hence, its activity.

## Discussion

The present study aims to define the relationship between HSulf-2 and ductal carcinoma *in situ *progression to invasive ductal carcinoma using the MCF10DCIS progression model. Although HSulf-2 has been reported to be up-regulated in breast cancer [23], its role in breast cancer progression has not been clearly defined. Here we utilized a unique cell line which expresses HSulf-2 and has the ability to form ductal lesions similar to those found in DCIS pathology in the human breast. By utilizing mouse mammary fat pad injections to evaluate the impact of HSulf-2 depleted MCF10DCIS cells on tumor growth, we found that HSulf-2 knockdown significantly attenuated tumor size, promoted apoptosis and retained comedo lesions for a longer period of time. It is notable that apoptosis was predominantly limited to the inner center or luminal area of comedo structures in HSulf-2 depleted xenografts. This indicates that loss of HSulf-2 selectively renders inner luminal cells of comedo lesions to undergo apoptosis presumably due to the tumor microenvironment resulting in culmination of the apoptotic program, which triggers spontaneous apoptosis in comedo lesions [33]. HSulf-2 loss up-regulated both the number and size of comedo structures with intact basement membrane. A striking feature of HSulf-2 depleted xenografts is the maintenance of the integrity of basement membrane even at later stages (Week 7) of DCIS to IDC progression, which suggests that HSulf-2 presence is essential for basement membrane disintegration. Basement membrane is a physical barrier between epithelial cells and stromal cells. Many MMPs (proteases) have been shown to play important roles in the remodeling of basement membrane and invasion of surrounding tissues [34]. Importantly, HSulf-2 silencing attenuated transition from DCIS to IDC by limiting MMP-9 expression and activities required for basement membrane degradation. Several members of the MMP family have been shown to be up-regulated prior to progression from DCIS to IDC in MCF10DCIS model [33]. Proteolysis of extracellular matrix proteins and basement membrane by these proteases results in the disruption of this barrier to promote invasion into surrounding stroma. The effect of HSulf-2 loss was specific to MMP-9, whereas no effect on MMP-2 was observed. MMP-9 has previously been shown to be a predominant matrix protease expressed in ductal lesions [7]. Our *in vivo *data show that HSulf-2 depletion markedly attenuates tumor growth. Supporting this notion, previous studies have identified HSulf-2 as one of the top 50 genes up-regulated in DCIS to IDC [25]. Similarly, in two different mouse models of mammary carcinoma, HSulf-2 up-regulation was associated with pro-angiogenic activity [23]. Our data provide a novel insight by raising the possibility that HSulf-2 may play an important role in the disintegration of basement membrane and promoting invasion of surrounding tissue. In addition to retention of comedo lesions even at Week 7 of tumor growth, HSulf-2 deficient xenografts were predominantly apoptotic. Massive apoptosis was evident in the center of comedo lesions and not near the basement membrane. This could be explained in several ways: a) it can be postulated that cells in the center of comedo lesions are often highly hypoxic and have a decreased supply of nutrients and b) these cells are separated from extracellular matrix protein of basement membrane and, hence, lack adhesion, and that HSulf-2 knockdown further sensitizes these cells to apoptosis due to lack of survival signals (growth factor and adhesion mediated). In other words, HSulf-2 depletion might pave way for luminal clearance in these comedo lesions as a result of apoptosis. Previous reports have also documented that HSulf-2 promotes cellular resistance to apoptosis in HCC cell lines [35]. Our study suggests that progression of DCIS to IDC might depend on HSulf-2 activities. Therefore, therapeutically targeting this enzyme either by shRNA or by a small molecule inhibitor may serve to improve our chances of controlling the progression of DCIS to IDC. Our data do not concur with a more recent study highlighting a tumor suppressor role of HSulf-2 in MDA231 cell line [24]. This study adequately addressed the role of HSulf-2 in the context of metastatic propensity of highly aggressive MDA231 cell line. However, caution should be exercised, as enhanced expression of HSulf-2 might promote nontargeted effects on tumor growth. Secondly, the specificity of substrates of HSulf-2 -HSPGs located at the cell surface could contribute to the differential response to the presence of HSulf-2 based on the binding affinity of specific HSPGS towards different growth factors. Thus, it is plausible that observed differences could partly depend on the nature of specific substrates (HSPGs) expressed in the different cell lines with HSulf-2 expression. Mechanistically, HSulf-2 has been shown to attenuate bFGF2 signaling but promotes Wnt signaling [36-38]. Activated Wnt signaling is common in mammary tumors despite lack of mutations in Wnt pathway genes [39]. Therefore, HSulf-2 presence may promote autocrine induction of Wnt signaling during breast tumorigenesis as previously reported [22].

In all, this is the first report which highlights the critical role of HSulf-2 in the progression of DCIS to IDC in MCF10DCIS cell line xenograft model. Validation of this finding in human tumors could lead to HSulf-2 as a biomarker of breast cancer progression. Additionally, we propose that therapeutic targeting of HSulf-2 could lead to improved clinical outcome in patients with breast cancer

## Conclusions

Silencing of heparan sulfatase 2 attenuates breast cancer growth and inhibits basement membrane disruption in a matrix metalloprotease dependent process.

## Abbreviations

BM: basement membrane; DAPI: 4',6-diamidino-2-phenylindole; DCIS: ductal carcinoma *in situ*; EMT: epithelial mesenchymal transition; HSPG: heparan sulfate proteogylcans; HSulf2: Heparan sulfatase 2; IDC: invasive ductal carcinoma; MMP: matrix metalloproteinase; NTC: non-target control; PBS: phosphate-buffered saline; PI: propidium iodide

## Competing interests

The authors declare that they have no competing interests.

## Authors' contributions

AK, HMK, HK and JM performed the experiments. LRR's lab generated the antibody against HSulf2. MPG, SHK and VS supervised the study.
